# The complete chloroplast genome sequence of *Elatostema stewardii* Merr. (Urticaceae)

**DOI:** 10.1080/23802359.2022.2108348

**Published:** 2022-08-18

**Authors:** Liang-Hai Yang, Yu-Qing Feng, Lu Ding, Long-Mei Tong, Zhe-Chen Qi, Xiao-Ling Yan

**Affiliations:** a College of Life Sciences and Medicine, Zhejiang Province Key Laboratory of Plant Secondary Metabolism and Regulation, Zhejiang Sci-Tech University, Hangzhou, China; b Shanghai Chenshan Plant Science Research Centre, Chinese Academy of Sciences, Shanghai Chenshan Botanical Garden, Shanghai, China

**Keywords:** *Elatostema stewardii*, Urticaceae, chloroplast genome, phylogenetic analysis

## Abstract

*Elatostema stewardii* is an important medicinal plant endemic to China. In this study, the complete chloroplast genome of *E. stewardii* was sequenced and assembled using next-generation sequencing technology. The complete chloroplast genome length of *E. stewardii* was 150,263 bp, including two inverted repeats (IRs) of 24,681 bp, which are separated by LSC and SSC of 83,791 bp and 17,110 bp, respectively. A total of 129 genes were included in the genome, consisting 85 protein-coding genes, eight rRNA genes, and 36 tRNA genes, the overall GC content of this genome was 36.3%. There are few studies on the genus *Elatostema* of Urticaceae, this chloroplast genome sequence will provide useful data for further research on solving the generic and familial relationships in Urticaceae.

*Elatostema stewardii* Merrill 1925 (Urticaceae) is a perennial plant native to China. It is commonly found in shady, damp areas near woodlands and streams. As a traditional Chinese medicine, *E. stewardii* promotes blood circulation, disperses silt, and has detumescence and detoxification effects. Its roots can be used to repair fractures, and its stems and leaves can be used to treat coughs (Yang et al. 2012; Tseng et al. 2019). Furthermore, *E. stewardii* is a popular indoor and outdoor ornamental plant. It is widely used as an excellent shade and humidity foliage ground cover plant. So far, chloroplast genomes of only three species have been reported in *Elatostema* (Fu et al. 2019; Wang et al. 2020; Fu et al. 2021). We assembled and characterized the complete chloroplast genome of *E. stewardii* for the first time in order to better understand the potential genetic information and phylogenetic relationship in *Elastostema* and Urticaceae.

The leaves of *E. stewardii* were collected from Xuancheng, Anhui, China (GPS: E 118°43′08.57″, N 30°35′52.01″). We used DNA Plantzol Reagent (Invitrogen, Carlsbad, CA) to extract DNA from silica dried leaves. The leaf samples and extracted DNA were stored at Zhejiang Province Key Laboratory of Plant Secondary Metabolism and Regulation, Zhejiang Sci-Tech University (http://sky.zstu.edu.cn) under the voucher number ZSTU01186 (collected by Zhe-Chen Qi and zqi@zstu.edu.cn). The sequencing libraries were prepared using Illumina's TruSeq Nano DNA Library preparation kit (350 bp median insertion) according to the manufacturer's protocol. Plastid sequences were generated using Illumina Hiseq 2500 platform (Illumina Inc., San Diego, CA). Overall, approximately 14.8 million high-quality clean reads (150 bp PE read length) were processed by Trimmomatic (Bolger et al. 2014). We used 14,793,667 reads to assemble the chloroplast genome by GetOrganelle (Jin et al. 2020), and annotated by GeSeq (Tillich et al. 2017) and GENEIOUS v11.1.5 (Biomatters Ltd., Auckland, New Zealand). Finally, manually checked and adjust to the chloroplast genome data.

The complete *E. stewardii* chloroplast sequence (GenBank accession no. MZ292972) has a total length of 150,263 bp, and consists of a large single-copy region (83,791 bp for LSC), a small single-copy region (17,110 bp for SSC), and two reverse repeat regions (24,681 bp for inverted repeat (IR)). The total GC content of *E. stewardii* chloroplast genome was 36.3%. The genome contains 129 genes (85 protein-coding genes, eight rRNA genes, and 36 tRNA genes). There were 17 genes with two copies, including six PCG genes (*ndhb*, *rps7*, *rps12*, *ycf2*, *rpl2*, and *rpl23*), seven tRNA genes (*trnl-CAU*, *trnl-CAA*, *trnv-GAC*, *trnl-GAU*, *trna-UGC*, *trnR-ACG*, and *trnN-GUU*), and all four rRNA species (*rrn16*, *rrn23*, *rrn4.5*, and *rrn5*). In the genome, 11 protein-coding genes (*rps16*, *atpF*, *rpoC1*, *petB*, *petD*, *rpl16*, *rpl2*, *ndhB*, *ndhA, ndhB,* and *rpl2*) contain one intron, and three protein-coding genes (*ycf3*, *clpP*, *rps12*) contain two introns.

We obtained chloroplast genome data of all three published *Elatostema* and 11 other Urticaceae species from NCBI GenBank to study the phylogenetic position of *E. stewardii* in Urticaceae. *Urtica lobatifolia* was used as outgroup for constructing the phylogenetic tree. The sequence alignment was conducted by MAFFT v7.450 (Katoh and Standley 2013), of which parameter used the default. Based on TVM + F+R3 model and 5000 bootstrap replicates, the maximum-likelihood (ML) analysis was performed by using IQTREE v2.0.6 (Nguyen et al. 2015). The result showed that *E. stewardii is* sister to a clade formed by *E. qinzhouense* (Figure 1). The complete chloroplast genome of *E. stewardii* will provide necessary genetic resource and background data for further phylogenetic study of the Urticaceae.

**Figure 1.**
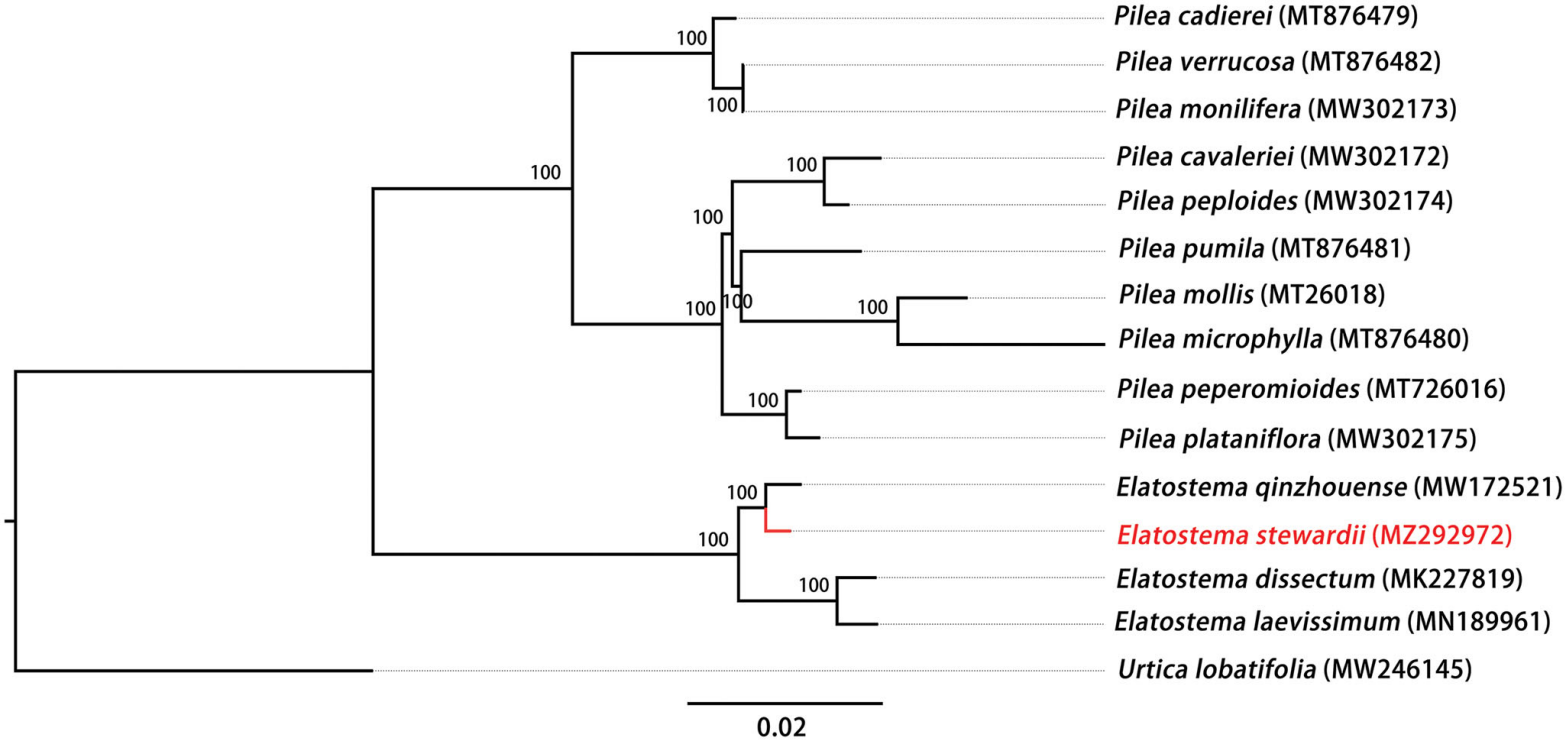
The phylogenetic tree based on the complete chloroplast genome of *Elatostema stewardii* and other 14 species, with *Urtica lobatifolia* as outgroup. Bootstrap values are shown on each node. Accession numbers are listed with each species.

## Data Availability

The genome sequence data that support the findings of this study are openly available in GenBank of NCBI (https://www.ncbi.nlm.nih.gov) under the accession no. MZ292972. The associated BioProject, SRA, and Bio-Sample numbers are PRJNA732525, SRR14638108, and SAMN19321915, respectively. The DNA matrix and phylogenetic tree that support the findings of this study are openly available in figshare at https://doi.org/10.6084/m9.figshare.18094190.v1.
